# Mitochondrial Adaptations in Elderly and Young Men Skeletal Muscle Following 2 Weeks of Bed Rest and Rehabilitation

**DOI:** 10.3389/fphys.2019.00474

**Published:** 2019-05-01

**Authors:** Alessia Buso, Marina Comelli, Raffaella Picco, Miriam Isola, Benedetta Magnesa, Rado Pišot, Joern Rittweger, Desy Salvadego, Boštjan Šimunič, Bruno Grassi, Irene Mavelli

**Affiliations:** ^1^Department of Medicine, University of Udine, Udine, Italy; ^2^Institute for Kinesiology Research, Science and Research Centre, Koper, Slovenia; ^3^Department of Pediatrics and Adolescent Medicine, University of Cologne, Cologne, Germany; ^4^Institute of Aerospace Medicine, German Aerospace Center (DLR), Cologne, Germany; ^5^Institute of Bioimaging and Molecular Physiology, National Research Council, Milan, Italy; ^6^INBB Istituto Nazionale Biostrutture e Biosistemi, Rome, Italy

**Keywords:** mitochondria-related proteins, immobility, aging, exercise, skeletal muscle, western blot, *in silico* gene expression data mining

## Abstract

The aim of the study was to evaluate the expression levels of proteins related to mitochondrial biogenesis regulation and bioenergetics in *vastus lateralis* muscle biopsies from 16 elderly and 7 young people subjected to 14 days of bed-rest, causing atrophy, and subsequent 14 days of exercise training. Based on quantitative immunoblot analyses, in both groups a reduction of two key regulators of mitochondrial biogenesis/remodeling and activity, namely PGC-1α and Sirt3, was revealed during bed-rest, with a subsequent up-regulation after rehabilitation, indicating an involvement of PGC-1α-Sirt3 axis in response to the treatments. A difference was observed comparing the young and elderly subjects as, for both proteins, the abundance in the elderly was more affected by immobility and less responsive to exercise. The expression levels of TOM20 and Citrate Synthase, assayed as markers of outer mitochondrial membrane and mitochondrial mass, showed a noticeable sensitivity in the elderly group, where they were affected by bed-rest and rehabilitation recalling the pattern of PGC-1α. TOM20 and CS remained unchanged in young subjects. Single OXPHOS complexes showed peculiar patterns, which were in some cases dissimilar from PGC-1α, and suggest different influences on protein biogenesis and degradation. Overall, exercise was capable to counteract the effect of immobility, when present, except for complex V, which was markedly downregulated by bed-rest, but remained unaffected after rehabilitation, maybe as result of greater extent of degradation processes over biogenesis. Phosphorylation extent of AMPK, and its upstream activator LKB1, did not change after bed-rest and rehabilitation in either young or elderly subjects, suggesting that the activation of energy-sensing LKB1-AMPK signaling pathway was “missed” due to its transient nature, or was not triggered under our conditions. Our study demonstrates that, as far as the expression of various proteins related to mitochondrial biogenesis/remodeling, adaptations to bed-rest and rehabilitation in the two populations were different. The impact of bed-rest was greater in the elderly subjects, where the pattern (decrease after bed rest and recovery following rehabilitation) was accompanied by changes of mitochondrial mass. Modifications of protein abundance were matched with data obtained from gene expression analyses of four public human datasets focusing on related genes.

## Introduction

Skeletal muscle is a very plastic tissue that responds and adapts quickly to inactivity or exercise. Around the fourth decade of life, skeletal muscle mass and functional performance, including oxidative metabolism, inevitably decline (Short et al., 2005; Salvadego et al., 2011; Dirks et al., 2014; Wall et al., 2014). Such decline accelerates with aging (Hughes et al., 2001) and it is usually associated with a decreased physical activity that can be deleterious for skeletal muscle, cardiovascular function, metabolic control and several other systems of the body (Brower, 2009). Physical inactivity in elderly people is a growing problem in western countries, also due to the impact of hospitalization. In fact, injuries in the elderly are very common and even a brief period of immobilization can result in a great loss of muscle mass and function, hard to restore even with rehabilitation interventions (Hvid et al., 2010).

Because of these clinical implications, the research on elderly subjects and their response to physical inactivity has been very active in the last few years. In this context, bed-rest studies are often employed, in order to simulate profound inactivity as well as microgravity. Bed-rest studies carried out by our group on young subjects observed a significant functional impairment of skeletal muscle oxidative metabolism evaluated *in vivo* following 10 days (Salvadego et al., 2016), 21 days (Salvadego et al., 2018), and 35 days (Porcelli et al., 2010; Salvadego et al., 2011) of bed-rest conditions. After 21 days, but not after 10 days of bed-rest, the functional impairment *in vivo* was associated with an impaired mitochondrial respiration evaluated *ex vivo*.

We discussed in detail in a recent publication (Pišot et al., 2016) previous bed-rest studies from different laboratories on elderly populations. In short, these studies often lacked a control group of young individuals, did not consider a rehabilitation phase, or did not comprehend periods of inactivity long enough to induce significant changes also in the young controls. These considerations prompted us to design a bed-rest study in which young (Y) and elderly (E) participants were exposed to precisely the same protocol of inactivity in bed for 14 days, and to a subsequent rehabilitation with high-intensity interval training for another period of 14 days. The details of the study, as well as some results about systemic variables, are given in Pišot et al. (2016) and Rejc et al. (2018). Briefly, adaptations to bed-rest and rehabilitation in the two populations were different. Interestingly, the impact of bed-rest on muscle mass and function (muscle force and power, fiber strength, and V’O_2_peak) was greater in E compared to Y, as well as the rehabilitation was slower and/or less complete in E.

There is still debate about which mechanisms are involved in the loss of muscle mass and function after inactivity. During the last few years interest has arisen around the role played by skeletal muscle mitochondrial function and biogenesis following inactivity and aging (Carter et al., 2015), as well as in the pathogenesis of inactivity-related diseases (Booth et al., 2012). Proteomic and gene expression analyses documented decreases in expression of peroxisome proliferator-activated receptor-γ coactivator-1α (PGC-1α) and OXPHOS complexes as consequence of induced disuse, in association with a reduction in mitochondrial biogenesis and an overall impairment of energy metabolism (Chen et al., 2007; Alibegovic et al., 2010a; Brocca et al., 2010, 2012; Ringholm et al., 2011). “Upstream” of PGC-1α, AMPK/LKB1 energy sensor signaling pathway has been reported to be involved (Ringholm et al., 2011; Brocca et al., 2012). AMPK, a serine/threonine protein kinase, has emerged as a master sensor of cellular energy balance in mammalian cells, including skeletal muscle, (Hardie and Sakamoto, 2006; Kjøbsted et al., 2018) and one of the upstream activators of AMPK signaling pathway is LKB1. In the context of mitochondrial activity-regulated signaling “downstream” of PGC-1α, Sirt3, has emerged as the major regulator of mitochondrial protein deacetylation (Lombard et al., 2007; Menzies and Auwerx, 2013).

The aim of the present study was to evaluate the changes occurred as response to inactivity and rehabilitation by E people, compared to Y, in the expression levels of the proteins above mentioned, playing a key role for mitochondrial biogenesis and function. In no previous studies, the expression levels of such proteins were determined in Y and E subjects undergoing the same duration of inactivity in bed followed by the same rehabilitation protocol, as in the present study. We hypothesized a pattern of changes in the protein expression similar to that described for systemic variables directly related to mitochondrial function (muscle mass and peak aerobic power) in the same subjects exposed to the same environmental stimuli, i.e., a more pronounced decrease during bed-rest in E vs. Y and a slower/incomplete rehabilitation.

Furthermore, with the aim to validate our protein expression data, we performed some *in silico* analyses of public human gene expression datasets, focusing in particular on mitochondria-related genes involved in skeletal muscle responses to immobility and rehabilitation in young adult individuals, or demonstrated to change in association with aging. Combining data from bioinformatics analyses of gene expression with those of protein abundance from our bed-rest study encouraged us to hypothesize possible molecular mechanisms implicated in the effects observed in Y and E subjects.

## Materials and Methods

### Participants

Twenty-three healthy men, of which 7 young (Y; aged 18–30 years) and 16 elderly subjects (E; aged 55–65 years) were recruited for the study. All participants underwent medical examination and routine blood and urine analysis. Basic anthropometric parameters of the two groups and exclusion criteria are reported previously in Pišot et al. (2016). Participants were informed of the purpose, procedures and potential risk of the study before signing the informed consent. The study was performed in accordance with the ethical standards of the 1964 Declaration of Helsinki and was approved by the National Ethical Committee of the Slovenian Ministry of Health on April 17, 2012 under the acronym: IR-aging 1200.

### Study Design

The study was conducted in concomitance with the study of Pišot et al. (2016) and Rejc et al. (2018) in a controlled medical environment at the Valdoltra Orthopedic Hospital, Slovenia. The participants were housed in standard air-conditioned hospital rooms and were under constant surveillance with 24-h medical care. For 14 days, the participants performed all daily activities in bed in the horizontal position and followed an individually controlled eucaloric diet. Such conditions are denoted as bed-rest (BR). Dietary energy requirements were designed for each subject by multiplying resting energy expenditure by factors 1.2 and 1.4 in BR and during the rehabilitation period, respectively (Biolo et al., 2008). The macronutrient food content was set at 60% of carbohydrates, 25% fat and 15% of proteins. Energy balance was checked weekly by fat mass assessment performed with bioelectrical impedance analysis (tetra-polar impedance-meter, BIA101, Akern, Florence, Italy), using the software provided by the manufacturer, as in Rejc et al. (2018). After the BR participants underwent a rehabilitation protocol (R+14) that consisted of 2-week supervised multimodal exercise program with 3 sessions per week as described in details in Rejc et al. (2018). In each session, participants performed 12-min warm-up, 15–20 min of balance and strength training and 20–30 min of aerobic training (high-intensity interval training).

Three different biopsies were taken from *vastus lateralis* muscle of each subject: one before starting bed-rest for BDC, one after the bed-rest period (BR14), the last after the rehabilitation protocol (R+14) and specifically 4–5 days after the final training session during the period where the subjects completed the *in vivo* performance tests.

### Procedures

#### Muscle Biopsies

Samples were obtained from the mid-region of the left *vastus lateralis* muscle. Biopsy was done after anesthesia of the skin, subcutaneous fat tissue, and muscle fascia with 2 ml of lidocaine (2%). A small incision was then made to penetrate skin and fascia, and the tissue sample was harvested with a purpose-built rongeur (Zepf Instruments, Tuttlingen, Germany). The samples put in cryopreservation solution were immediately frozen in liquid nitrogen, and stored at -80°C until the analyses (Kuznetsov et al., 2003).

#### Immunoblot Sample Preparation

Just thawed biopsy samples were rapidly washed in PBS solution, dried, weighted and placed in a cooled 2 mL glass Teflon Potter-Elvehjem (Wheaton, IL, United States), in a suspension 1:4 w/v with PBS containing 0.32 M sucrose, P8340 – Sigma protease inhibitors (1:50 v/v) and 10 mM NaF + 1 mM Na_3_VO_4_ as phosphatase inhibitors (Mavelli et al., 1978). Samples were homogenized with 40 motor driven strokes (ForLab AT120, Carlo Erba, Italy) and aliquots were withdrawn and stored at -80°C. Further 1:1 v/v dilution of the residual homogenates was made with RIPA buffer 2× (300 mM sodium chloride, 2% NP-40, 1% sodium deoxycholate, 0.2% SDS, 0.8 mM EDTA and 100 mM Tris, pH 8.0), followed by other 40 motor driven strokes, to obtain a better membrane protein solubilization, and 30 min of incubation. All processes were carried out on ice-bath. Homogenates were centrifuged at 10,000 ×*g* for 10 min and the extracts were stored at -80°C until using for the assays. Protein concentration was tested with Lowry assay (Lowry et al., 1951) using BSA as a standard.

Extracts of H9c2 cell line (ATCC^®^ CRL-1446^TM^) were obtained by resuspension of cellular pellet (10^6^ cells/ml) in RIPA buffer (150 mM sodium chloride, 1% NP-40, 0.5% sodium deoxycholate, 0.1% SDS, 0.4 mM EDTA and 50 mM Tris, pH 8.0), containing protease (P8340 - Sigma) and phosphatase (10 mM NaF and 1 mM Na_3_VO_4_) inhibitors. After 30 min of incubation at 4°C, the samples were centrifuged at 14,000 ×*g* for 15 min at 4°C. Extracts were used as internal standards (IS) for quantification of immunoblot results.

#### Quantitative Immunoblot

Separation of sample’s different proteins was obtained by electrophoresis in denaturing conditions (SDS-PAGE), following Laemmli’s method (Laemmli, 1970) with Tris-glycine running buffer, using 8–16% polyacrylamide gradient precast resolving gels (Thermo Fisher Scientific, Waltham, MA, United States). After separation, analysis of the protein expression levels was carried out by immunoblotting. After the proteins were electro-transferred from the gel to a PVDF membrane, the membrane was divided based on the molecular weight of the single proteins according to the molecular weight markers. Each part of the membrane was blocked in a Tris-Buffered Saline (TBS) solution, containing 0.1% Tween 20, 2.5% BSA solution for 1 h and then incubated overnight with the proper antibodies. Specifically, the membranes were probed with antibodies vs. AMPK and p-AMPK^Thr172^ 1:500 (Santa Cruz, Dallas, TX, United States, catalog numbers sc-74461 and sc-33524, respectively), GAPDH 1:10000 (Santa Cruz, sc-32233), CS 1:10000 (AbCam, Cambridge, United Kingdom, ab 129095), PGC-1α 1:5000 (AbCam, ab 722301), Sirt3 1:1000 (CST, Danvers, MA, United States, 5490S), LKB1 and p-LKB1^Ser482^ 1:1000 (Phosphoplus Duet CST, 5132) and TOM20 1:7000 (Santa Cruz, sc-17764). We also probed the membranes with AbCam Ab Cocktail (ab 110413) vs. OXPHOS complexes (1:5000) for complex I (mitochondrial NADH dehydrogenase [ubiquinone] 1 beta subcomplex subunit 8 – NDUFB8), complex II (succinate dehydrogenase [ubiquinone] iron-sulfur subunit B – SDHB), complex III (Cytochrome b-c1 complex subunit 2 – UQCRC2), complex V (CV subunit α – ATP5A1). For complex IV a single antibody against cytochrome c oxidase subunit 4 – COXIV (Abcam, ab 110272, 1:10000) was used due to its greater efficiency. Thereafter, the membranes were incubated in the presence of the proper secondary antibody (rabbit-anti-mouse [61–6520] or goat-anti-rabbit [32460], Thermo Fisher Scientific) conjugated with horseradish peroxidase (HRP).

For quantification purposes, each gel was loaded with 11 samples along with molecular weight markers (Bio-Rad Laboratories, Berkeley, CA, United States) and IS (prepared as described above) in order to normalize the results from the single gels with those of replicates or different samples. 20 μg of proteins were loaded for each sample and 40 μg of IS. Samples from both Y and E subjects were assayed together matching the conditions (namely all BDC or BR14 or R+14 samples).

The protein bands were visualized by an enhanced chemiluminescence method using ChemiDoc (Bio-Rad Laboratories) and quantified with ImageQuant TL program (GE Healthcare, Little Chalfont, United Kingdom). Quantification was made based on “Adjusted Volume Intensity,” i.e., the volume given by the sum of the intensities of the pixels inside the boundary volume corrected for the background. The intensity of each band was normalized on total bands revealed by Coomassie-staining of PVDF membrane, considered as appropriate loading and transferring control (Welinder and Ekblad, 2011). Each sample was tested in triplicate and values were expressed as Arbitrary Units (AU).

**FIGURE 1:**
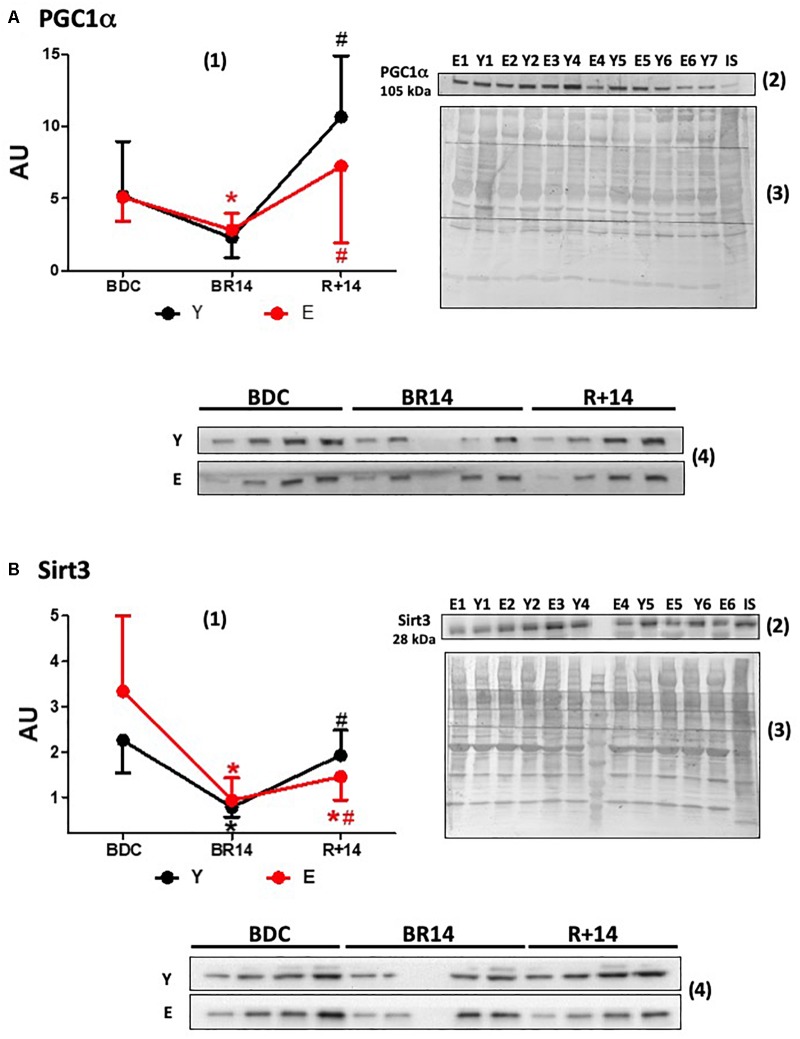
Changes in expression levels of PGC-1α **(A)** and Sirt3 **(B)** in *vastus lateralis* muscle biopsies from young and elderly subjects after bed rest and rehabilitation. Panels **(A,B)** graphs denoted as (1) represent immunoblot data of 7 young (Y; aged 18–30 years) and 16 elderly (E; aged 55–65 years) subjects, under the three conditions: BDC, baseline data collection before bed rest; BR14, after 14 days bed rest; R+14, after 2-week rehabilitation. Gels were loaded with samples from Y and E subjects (20 μg of proteins) along with IS (40 μg), prepared as described in Section “Materials and Methods.” ImageQuant TL values for single immunoreactive bands normalized to Coomassie staining of PVDF membrane and corrected for IS are expressed as arbitrary units (AU). Quantitative data are means ± SD of three different assays for each subject. Red line: Elderly group; black line: Young group. ^∗^Represents statistical significance (*p* < 0.05) vs. BDC condition, # between BR14 and R+14 conditions. Immunoblot images denoted as (2) are representative of experiments with samples from both Y and E subjects loaded on each gel matching the conditions (namely all BDC or BR14 or R+14 samples), and images denoted as (3) are representative of the corresponding Coomassie-stained whole PVDF membranes, used as loading and transferring measurement. Immunoblot images denoted as (4) are representative of experiments where each subject was analyzed individually by loading gel with samples of the three investigated conditions together (BDC, BR 14, and R+14). Gels were loaded with scalar amounts (5, 10, 15, and 20 μg) of Y or E samples to verify the linearity of the band intensity vs. the loaded protein. The central lane of the gel was loaded with molecular weight markers.

In separate experiments, the single subjects were also analyzed individually by loading each gel with samples of the three investigated conditions (BDC, BR 14, and R+14). Gels were loaded with scalar amounts (5, 10, 15, and 20 μg) of Y or E samples, to verify the linearity of the band intensity vs. the loaded protein amount. As an example, see representative immunoblot images denoted as (4) of Figure 1.

#### Statistical Analysis

Continuous variables were summarized as mean ± standard deviation. Data were tested for normal distribution using Shapiro–Wilk test. Equality of variance was assessed using Levene test. The significance of differences of expression levels of analyzed proteins between groups (Y vs. E) throughout different conditions (BDC or BR 14 or R+14) was explored using the Linear Mixed Models for Longitudinal Data. Comparisons between groups for each condition were performed using *t*-test. Comparisons among the different conditions within each group were achieved using paired *t*-test. Bonferroni correction for multiple comparisons was applied.

### Gene Expression Analysis

Human microarray datasets were downloaded manually from public repositories, ArrayExpress (Parkinson et al., 2007) and GEO (Barrett et al., 2011); the data were all related with Bed-rest or atrophy due to clinical-associated disuse, as well as with muscle and aging. The raw files were downloaded when available. All the CEL files were processed together by using standard tools available within the affy package in R (Gautier et al., 2004). An UniGene ID centered Chip Description file (CDF) was used in order to have only one intensity value per gene. CDF was downloaded from the Molecular and Behavioral Neuroscience Institute Microarray Lab^1^ (Dai et al., 2005). All annotation information were downloaded from the same website. The normalization step was done with the standard RMA algorithm (Irizarry et al., 2003). For determination of the DEG Standard *t*-test was performed. Lists of the top DEG are in Supplementary Tables S1–S4 where the genes were selected based on fold change (>+1.5 or <-1.5; >+1.3 or <-1.3) and *p* < 0.05. *Gene enrichment* analysis on each DEG list was performed using DAVID 6.8 software (Sherman et al., 2007).

As concern data related with muscle and aging, the Spearman’s correlation analysis was also performed making minimal assumptions about the relationship between the two diverse variables. Spearman’s Rank correlation coefficient was used to evaluate the strength and direction (negative or positive) of a relationship between two variables. Genes with a significant negative strong correlation were selected, correlation coefficient < -0.6, Benjamini–Hochberg corrected *p* < 0.05 (Supplementary Table S5). Rule of thumb for interpreting the size of a correlation coefficient was used. *Gene enrichment* analysis was then performed using DAVID 6.8 software.

## Results

All participants were able to comply with the study protocol. No dropouts and no medical complications occurred (see Pišot et al., 2016 for more details). Anthropometric, metabolic and muscle function data of the cohort were described in that publication (Pišot et al., 2016).

### Key Proteins of Mitochondrial Biogenesis and Function

Data about the expression of PGC-1α, a master regulator of mitochondrial biogenesis and structural/functional integrity, are given in Figure 1A. At the BDC, before the bed-rest campaign, PGC-1α protein levels were not different in E vs. Y. Both in E and in Y bed-rest (BR14) induced a remarkably similar decline in PGC-1α expression levels, although significance was not reached in the Y group. The subsequent rise following rehabilitation was less pronounced in E (R+14 values: 2.6 times vs. BR14) than that observed in Y (R+14 values: 4.7 times vs. BR14). Both in Y and in E PGC-1α levels “rebounded” after rehabilitation attaining in R+14 values higher than those observed at BDC. This rebound was more pronounced in Y (2 times vs. BDC levels compared to 1.4 in E).

The expression levels of Sirt3, the most characterized sirtuin in mitochondria, declined during bed-rest following a pattern similar to that of PGC-1α (Figure 1B), in accordance with the concept that they are controlled by PGC-1α in the nucleus (Brenmoehl and Hoeflich, 2013). No rebound to values over BDC was observed following rehabilitation in Y or in E. The recovery to the BDC levels after rehabilitation was complete in Y, whereas it was only partial in E.

**FIGURE 2:**
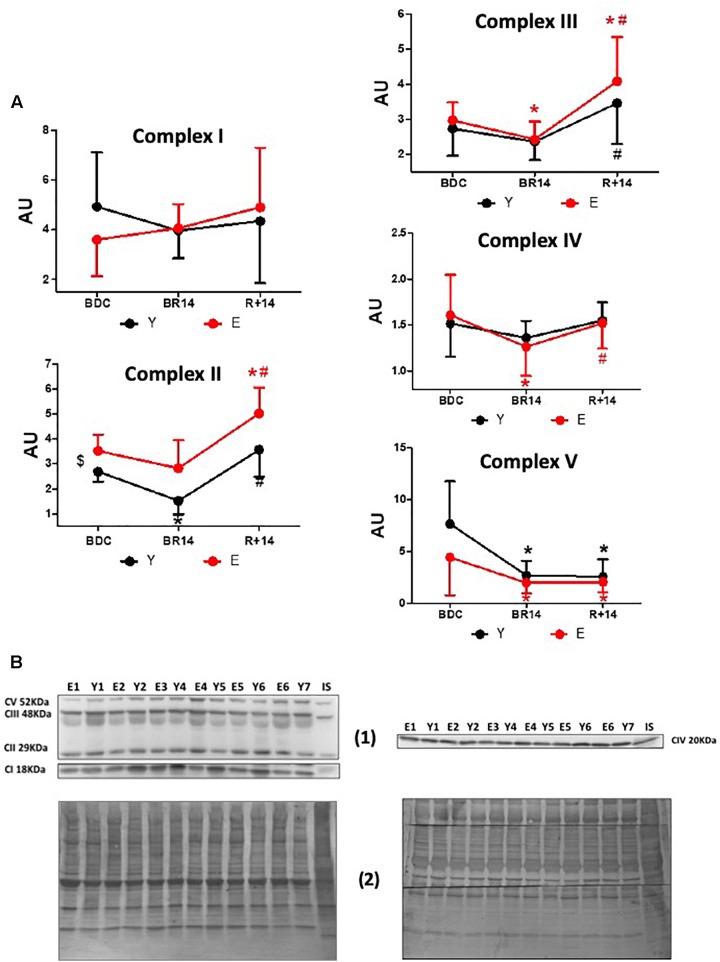
Changes in OXPHOS complexes’ expression levels in *vastus lateralis* muscle biopsies from young and elderly subjects after bed rest and rehabilitation. **(A)** Graphs represent immunoblot data (means ± SD) of OXPHOS complexes I, II, III, IV, V for the Y and E subjects, under BDC, BR14, and R+14 conditions. Red line: Elderly group; black line: Young group. **(B)** Immunoblot images denoted as (1) are representative of experiments with samples from both Y and E subjects loaded on each gel matching the conditions, along with IS, and images denoted as (2) are representative of the corresponding Coomassie-stained whole PVDF membranes used as loading and transferring measurement. All details of the analysis and quantification are as in Figure 1.

The effects of bed-rest and rehabilitation on OXPHOS complexes protein expression are shown in Figure 2. The behavior of the different complexes was rather heterogeneous. The respiratory chain carriers CII, CIII, and CIV showed both in Y and in E a similar general pattern, although in some cases the differences did not reach statistical significance. Namely, a decrease at BR14 and a recovery (in CII, CIII, and CIV) with a rebound (only in CII and CIII) at R+14 were observed, resembling the pattern described above for PGC-1α. At BDC, protein abundance of CII was significantly greater in E vs. Y, whereas for the other complexes no significant differences between groups were observed. Protein abundance of the respiratory chain carrier CI did not significantly change in any condition, both in Y and in E. Lastly, OXPHOS Complex V (CV) showed a unique pattern: both in Y and in E, the expression decreased at BR14 and did not recover at R+14.

**FIGURE 3:**
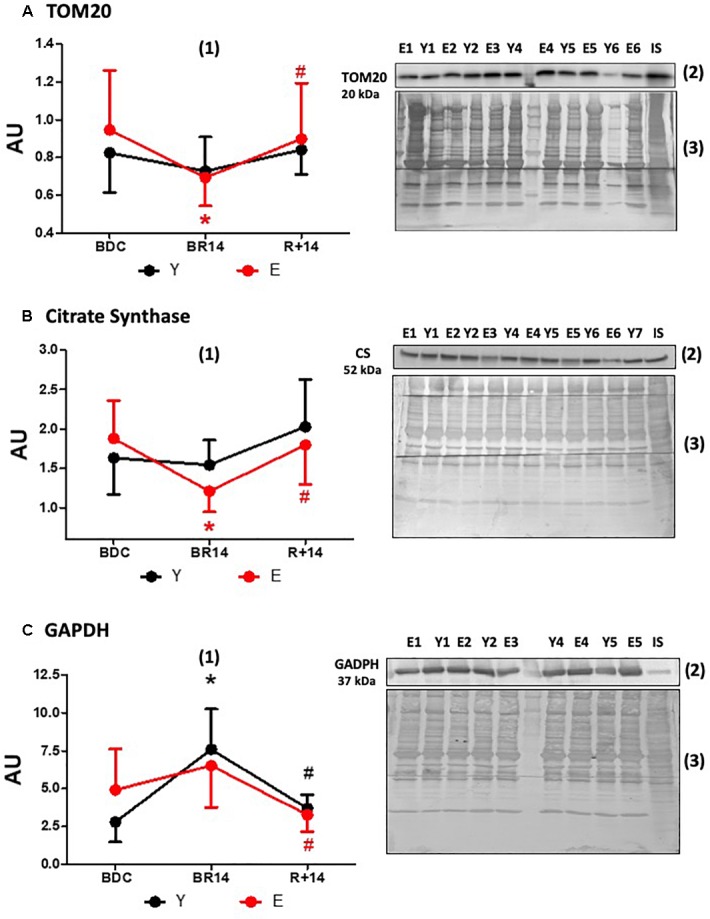
Changes in TOM20 **(A)**, Citrate Synthase **(B)**, and GAPDH **(C)** expression levels in *vastus lateralis* muscle biopsies from young and elderly subjects after bed rest and rehabilitation. Panels **(A–C)** graphs denoted as (1) represent immunoblot data (means ± SD) for the Y and E subjects under BDC, BR14, and R+14 conditions. Red line: Elderly group; black line: Young group. Immunoblot images denoted as (2) are representative of experiments with samples from both Y and E subjects loaded on each gel matching the conditions, along with IS, and images denoted as (3) are representative of the corresponding Coomassie-stained whole PVDF membranes, used as loading and transferring measurement. All details of the analysis and quantification are as in Figure 1.

In order to estimate mitochondrial mass we determined the expression levels of TOM20, a component of the translocase of the outer mitochondrial membrane, and CS, an enzyme of the mitochondrial matrix. The patterns of TOM20 (Figure 3A) and CS (Figure 3B) protein abundance were similar and appeared to be in agreement with those of PGC-1α and Sirt3 only in E, showing a decrease following bed-rest with a restoration following rehabilitation. Indeed, in E the levels were lower in BR14 than BDC and increased after rehabilitation (higher values in R+14 vs. BR14). The recovery vs. BDC was complete (no significant differences between R+14 and BDC). Conversely, in Y no statistically significant changes were observed.

The expression levels of the key glycolytic enzyme GAPDH are shown in Figure 3C. It should be pointed out that the change from BDC to BR14 in E was less pronounced than Y and did not reach the statistical significance. Nevertheless, in both groups the pattern was similar: the levels increased during bed-rest (suggesting a shift from oxidative to non-oxidative metabolism) and returned to BDC levels during rehabilitation.

**FIGURE 4:**
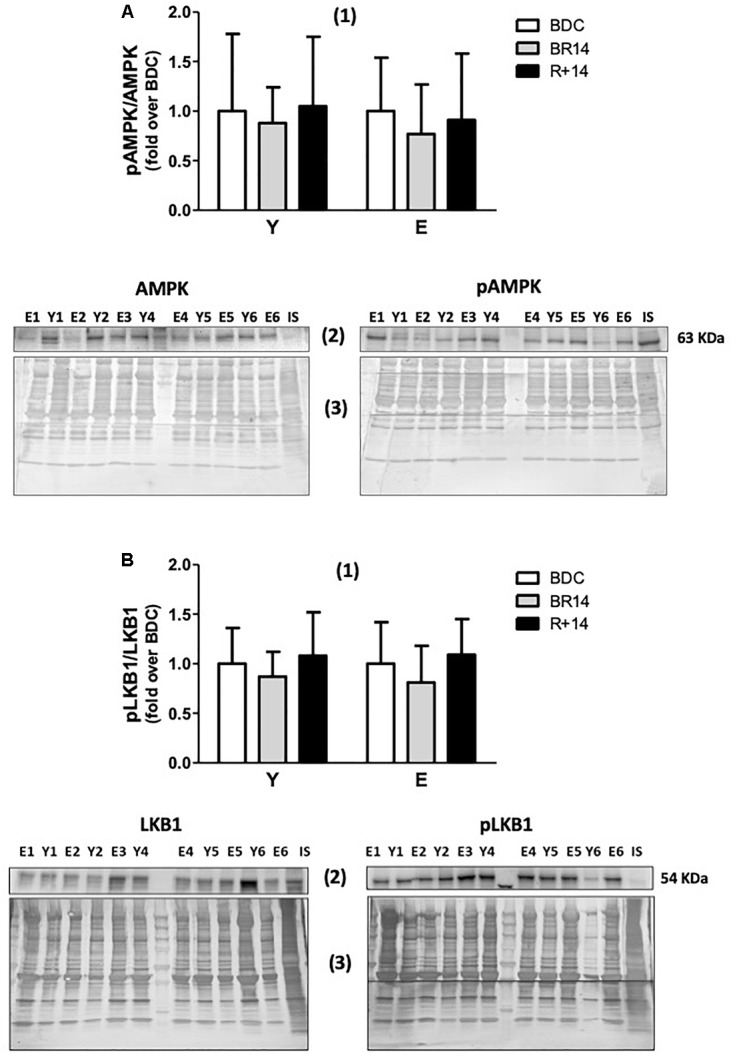
p-AMPK/AMPK **(A)** and p-LKB1/LKB1 **(B)** ratios in *vastus lateralis* muscle biopsies from young and elderly subjects after bed rest and rehabilitation. In both panels **(A,B)**, histograms denoted as (1) represent the fold-increase (means ± SD) of the normalized ratio between phosphorylated and total proteins from immunoblot analyses for Y and E subjects under BDC (empty columns), BR14 (gray columns), and R+14 (black columns) conditions. Immunoblot images denoted as (2) refer to total (on the left) and phosphorylated (on the right) proteins and are representative of experiments where samples from both Y and E subjects were loaded together on each gel matching the conditions, along with IS. Images denoted as (3) are representative of the corresponding Coomassie-stained whole PVDF membranes, used as loading and transferring measurement. All details of the analysis and quantification are as in Figure 1.

Finally, we also investigated the activation of the energy sensor AMPK, in order to evaluate if the observed changes were associated/driven by a condition of energy stress, and if AMPK signaling pathway, upstream of PGC-1α, was triggered in concert with the PGC-1α-Sirt3 axis. With this aim, we assessed the p-AMPK^Thr172^/AMPK ratio. Intriguingly, no significant changes were observed in the ratio (Figure 4A), as well as in the total AMPK protein levels (data not shown), in both groups across conditions. We also assessed the p-LKB1^Ser482^/LKB1 ratio considering that LKB1 is one of the upstream activators of AMPK signaling. No significant changes were observed even for such variable (Figure 4B), supporting the unexpected absence of AMPK activation.

### Gene Expression Data Mining From Public Datasets

In an attempt to validate our protein expression data, we performed some *in silico* analyses of public human gene expression datasets, focusing on mitochondria-related genes relevant for our bed-rest study. As no public databases were available relative to elderly people in immobility conditions, our analysis was focused on GEO-included datasets of genes involved in skeletal muscle responses to aging or to immobility/rehabilitation in young adult individuals.

Firstly, we analyzed GSE24215, which is one of the most complete dataset regarding the inactivity-induced responses of gene expression in adult healthy subjects along with the effects on exercise rehabilitation (Alibegovic et al., 2010b). Our DEG analysis focused on genes relevant for skeletal muscle structure/function with particular attention to mitochondria and energy metabolism, and showed that a number of such genes were significantly downregulated following bed-rest conditions resembling those of our study (list of the top DEG following inactivity in Supplementary Table S1). We present our results of *gene enrichment* analysis in Table 1 reporting the significantly enriched categories/terms, and summarize in Table 2 the DEG relative to such categories/terms (see gray cells) for the genes most relevant for our bed-rest study. Notably, among the downregulated mitochondrial genes, 2 are subunits of TIM and TOM complexes, 8 are subunits of OXPHOS Complexes I, II, IV, and V, and 1 is a subunit of permeability transition pore PTP. Interestingly, PGC-1α also results downregulated (PPARGC1A gene is included in category/term “hsa04920:adipocytokine signaling pathway”). Concerning the genes contained within the category/term “muscle proteins” (not shown in Table 2), it should be underlined that, along with 8 genes downregulated, 6 genes are upregulated including two myosin heavy chain in accordance to the recognized switch from slow to fast muscle fibers after immobility (Schiaffino and Reggiani, 2011; Lynch et al., 2015). After rehabilitative exercise training a number of mitochondria-related genes were found significantly upregulated with respect to immobility (list of the top DEG following post-inactivity exercise in Supplementary Table S2). Among these genes, much corresponding to those downregulated by bed-rest, there are subunits of mitochondrial proton-transporting ATP synthase Complex V and PTP, as well as of Complex IV, Complex II, TIM and TOM complexes. All are comprised within the 41 genes included in category/term “Mitochondrion” of *gene enrichment* analysis reported in Table 1 and are summarized in Table 2. Intriguingly, the categories/terms “hsa00190:Oxidative phosphorylation,” “GO:0005753∼mitochondrial proton-transporting ATP synthase complex,” and “hsa04920:adipocytokine signaling pathway” were not significantly enriched after the exercise training. This might be due to the higher number of genes upregulated after rehabilitation with respect to those downregulated after immobility (386 vs. 252). Of note, the category/term “Energy production and conversion” and GPD2 gene comprised within, appeared significantly downregulated after exercise, in agreement with a switch toward OXPHOS (Tables 1, 2). Indeed, GPD2 encodes for GPD2, which catalyzes the conversion of glycerol-3-phosphate using FAD as acceptor of reducing equivalents within the inner mitochondrial membrane.

**Table 1:** Differentially expressed genes after inactivity and subsequent exercise for muscle- and mitochondria-related proteins in *vastus lateralis*. GSE24215 dataset.

Category	Term	Count	%	*p*	Benjamini	Fold enrichment
**Gene enrichment analysis of downregulated genes after inactivity**						
UP_KEYWORDS	Mitochondrion	49	20.94	1.5E-16	3.14E-14	4.00
UP_KEYWORDS	Muscle protein	8	3.42	5E-06	0.00036	11.74
KEGG_PATHWAY	hsa00190:Oxidative phosphorylation	8	3.42	0.00303	0.03573	4.12
KEGG_PATHWAY	hsa04920:Adipocytokine signaling pathway	7	2.99	0.00049	0.01087	6.84
GOTERM_CC_DIRECT	GO:0005753∼mitochondrial proton-transporting ATPsynthase complex	4	1.71	0.00200	0.05541	15.62
**Gene enrichment analysis of upregulated genes after inactivity**						
UP_KEYWORDS	Muscle protein	6	1.78	0.00296	0.08000	6.10
**Gene enrichment analysis of downregulated genes after exercise**						
COG_ONTOLOGY	Energy production and conversion	4	3.15	0.00086	0.00257	18.33
**Gene enrichment analysis of upregulated genes after exercise**						
UP_KEYWORDS	Mitochondrion	41	11.39	7.3E-06	0.00031	2.15

*Category, original database/resource where the term orient; term, enriched terms associated with the gene list; count, genes involved in term; %, percentage of genes involved/total genes; p, modified fisher exact p-value, EASE Score; Benjamini, statistical correction; fold enrichment, down (up) regulated genes in a specific category/term over total down (up) regulated genes (%)**/**number of genes in that category/term over total number of genes (%). Lists of the top DEG in Supplementary Tables S1, S2. For DEG analysis, see Supplementary Table legends*.

**Table 2:** A summary of results obtained for mitochondria-related genes from DEG, correlation analysis, and *gene enrichment* of two public datasets.

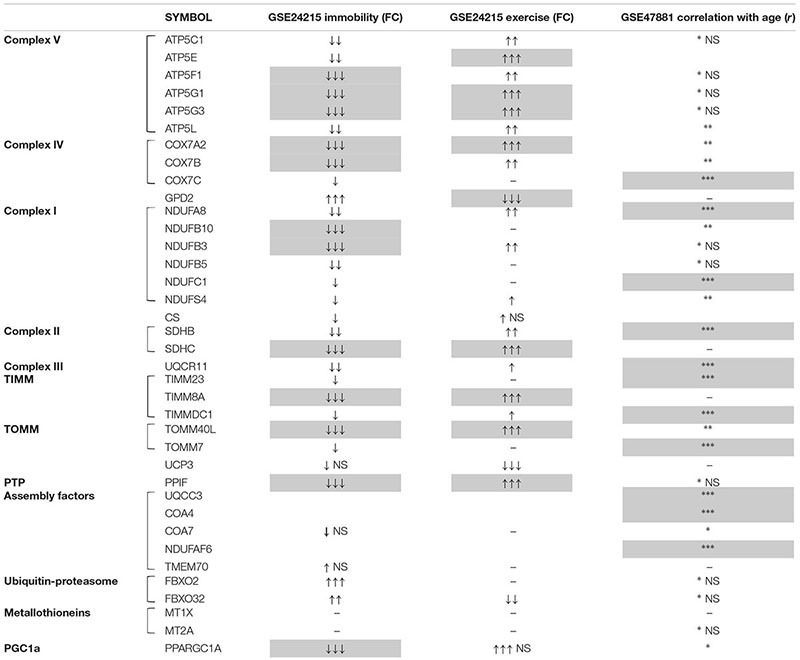

*(↑↑↑) FC > 1.5; (↑↑) 1.30 < FC < 1.49; (↑) 1.20 < FC < 1.29; (–) FC < 1.19.*

*(↓↓↓) FC < -1.5; (↓↓) -1.49 < FC < -1.30; (↓) -1.29 < FC < -1.20; (-) FC > -1.19.*

*(^∗∗∗^) negative strong correlation; (^∗∗^) negative moderately strong correlation; (^∗^) negative weak correlation; (-) r < -0.2 (no correlation). Empty cells: values not present in the array. The gray cells indicate data referred to results of the gene enrichment analyses (Tables 1, 3)*.

Remarkably, negligible changes were observed for CS either after bed-rest or after post-immobility exercise (Table 2), in accordance with the results of protein abundance obtained for the young group of our bed-rest study, where changes were observed for PGC-1α – Sirt3 and single OXPHOS complexes, but not for markers of mitochondrial mass.

Finally, consistent with the idea that protein turnover could be affected *via* alteration of breakdown pathways together with biogenesis, we also searched for genes involved in ubiquitin-proteasome pathway. Results of DEG analysis revealed that gene expression for FBXO32 (Atrogin-1) was significantly up- and down-regulated by immobility and rehabilitation, respectively, although just below the threshold chosen to define the top DEGs (Table 2 and Supplementary Tables S1, S2). Intriguingly FBXO2, that is another component of the ubiquitin E3 ligases playing important roles in the ubiquitin-proteasome protein-degradation pathway, resulted more markedly upregulated by immobility, but not affected following exercise.

We further analyzed the GEO dataset GSE8872 (Chen et al., 2007), including data from *medial gastrocnemius* muscle of adult subjects (around 30 years old) undergoing disuse atrophy due to shorter (5 days) immobilization attained using a short leg cast with the ankle in a neutral position.

From DEG analysis, performed with the aim to see if there were changes in mitochondria-related genes’ expression as an early response to muscle inactivity, resulted some interesting data, which we considered relevant for our bed-rest study (list of the top DEG in Supplementary Table S3). Not many subunits of only two OXPHOS complexes were downregulated significantly (Complex I NDUFS4/B3/B5 and Complex V ATP5G3/L/C1: ctrl/immobility FC = 1.49–1.62 around the threshold, *p* < 0.05). Notably, among the few genes resulted to be significantly upregulated there are UCP3 (uncoupling protein 3 – mitochondrial proton carrier), showing a ctrl/immobility FC = -1.51, *p* = 0.0055, as well as MT1X and MT2A, codifying for metallothioneins 1X and 2A (ctrl/immobility FC = -1.83 and -1.78, *p* = 0.014 and 0.001, respectively).

As a final point, to explore the expression of mitochondria-related genes involved in response to aging we focused on two GEO datasets comprising human microarray data from *vastus lateralis* biopsies of subjects with different age. Specifically, we analyzed the dataset GSE9103 (Lanza et al., 2008) for gene expression changes between young (18–30 years) and aged (58–76 years) sedentary people, as well as the dataset GSE47881 (Phillips et al., 2013) in order to perform a correlation analysis with age taking advantage from data of tree different groups of sedentary subjects (age 20–28, 45–55, and 64–75 years). For both datasets, the ranges of age analyzed included the age of the subjects of our bed-rest study (E: 55–65 years vs. Y: 18–30 years).

From DGE analysis of GSE9103 (list of the top DEG in Supplementary Table S4) emerged that some of the genes significantly downregulated in elderly subjects are relevant in the context of the proteins analyzed in our bed-rest study. Specifically, a marked decline was observed for the expression of TOMM40L (FC = -1.54; *p* = 0.0016), a gene encoding for the channel-forming subunit of the translocase of the outer mitochondrial membrane, which might be indicative of reduction of mitochondrial mass/biogenesis in elderly people. Conversely, negligible changes were observed for all OXPHOS complexes, while for PPARGC1A the decline was just below the threshold selected (FC = -1.3989; *p* = 0.0080). Finally, though of different extent, the downregulation of gene expression for TMEM70 (FC = -1.73; *p* = 0.0402) and COA7 (FC = -1.39; *p* = 0.0061), is also interesting with respect to our bed-rest study, as such genes are required for assembly of mitochondrial ATP synthase complex V and respiratory chain complex I and complex IV, respectively.

Notably, the results of the correlation analysis with age of mitochondria-related genes’ expression from the second dataset GSE47881indicate that there is a negative strong correlation with age of the expression of a number of mitochondrial genes relevant in the context of the proteins analyzed in our bed-rest study. The results of *gene enrichment* analysis are recapitulated in Table 3, while DEG are summarized in Table 2 (list of the top DEG in Supplementary Table S5). Specifically, negative strong correlation with age emerged for some subunits of respiratory carriers and assembly factors, as well as for some subunits of TIM and TOM complexes, which are included within the three categories/terms reported in Table 3 as highly significantly enriched. In addition, PGC-1α (PPARGC1A) showed a weak but significant negative correlation with age, while no correlation resulted for any subunits of mitochondrial ATP synthase complex V (Table 2).

**Table 3:** Mitochondria-related genes negatively correlated with age in *vastus lateralis*. GSE47881 dataset.

Category	Term	Count	%	*p*	Benjamini	Fold enrichment
**Gene enrichment analysis of genes negatively correlated with age (Spearman correlation analysis)**						
GOTERM_CC_DIRECT	GO:0005743∼mitochondrial inner membrane	32	7.27	5.5E-09	1.99E-06	3.42
UP_KEYWORDS	Mitochondrion	50	11.36	1.97E-07	2.19E-05	2.24
UP_KEYWORDS	Transit peptide	26	5.91	0.0000819	0.00679	2.43

*Rule of thumb for interpreting the size of a correlation coefficient was used. Genes with a significant negative strong correlation were selected (correlation coefficient -0.6 ≤ r ≤-0.8), Benjamini–Hochberg corrected p-values < 0.05. Category, original database/resource where the term orient; term, enriched terms associated with the gene list; count, genes involved in term; %, percentage of genes involved/total genes; p, modified Fisher Exact p-value, EASE Score; Benjamini, statistical correction; fold enrichment, down (up) regulated genes in a specific category class over total down (up) regulated genes (%)**/**number of genes in that category class over total number of genes (%). Lists of the top negatively correlated genes in Supplementary Table S5*.

## Discussion

The main aim of the present study was to evaluate variables related to mitochondrial biogenesis and function in young (Y) and elderly (E) subjects undergoing 14 days of profound inactivity (bed-rest), followed by 14 days of rehabilitation by a multimodal exercise program with an aerobic phase consisting in high-intensity intervals training. More specifically, we intended to compare the changes of the expression levels of key proteins related to the regulation of mitochondrial energy metabolism with those of “systemic” variables of functional evaluation, determined in the same subjects and recently published (Pišot et al., 2016; Rejc et al., 2018). The study was conducted under strictly controlled conditions and the period of inactivity was long enough to induce a marked muscle atrophy in both groups. Indeed, a significant decrease in quadriceps muscle volume occurred in elderly (-8.3%, *p* < 0.001), and the same trend was observed in the young controls (-6.1%, *p* = 0.052) (Pišot et al., 2016). We expected that the expression of the investigated proteins would be at the base of the functional adaptations occurring in skeletal muscles following inactivity and subsequent rehabilitation, supporting the role of mitochondrial regulation in muscle plasticity even in older individuals. The general finding of the previous study (Pišot et al., 2016) was that “systemic” variables of functional evaluation were often affected by inactivity more profoundly in E, in whom the rehabilitation was also less complete vs. that of Y, or did not occur. A similar pattern was observed for some variables determined in the present study. The main difference between the results of the two studies relates to baseline values, which in the present study were, in most cases, not significantly different in E vs. Y. As an example, the similar values observed for PGC-1α protein expression levels are in accordance with previous reports (Lanza et al., 2008; Irving et al., 2015). What observed for the systemic variables by Pišot et al. (2016) is in sharp contrast. For example, peak pulmonary O_2_ uptake (V’O_2_peak), a variable estimating maximal aerobic power, which should be related to mitochondrial function, was at baseline about 30% lower in E vs. Y (Pišot et al., 2016). In other words, a clear dissociation was present at baseline between systemic and mitochondrial variables related to oxidative metabolism, confirming the concept that mitochondrial factors are not the main determinant of systemic maximal aerobic power, and that factors “upstream” of mitochondria (mainly cardiovascular O_2_ delivery) are more relevant in this respect (Lundby et al., 2017). In the present study, most variables related to mitochondrial oxidative metabolism decreased following bed-rest. For some variables the decrease was more pronounced (or was statistically significant only) in E. A complete restoration was observed in Y for most mitochondrial variables; the restoration was incomplete in E in some cases. Thus, we infer that mitochondrial adaptations occurring under conditions of inactivity-induced atrophy and after rehabilitation went substantially in parallel with changes of systemic variables related to mitochondrial function.

### PGC-1α and Sirt3

In accordance with a decreased need of new mitochondrial proteins and energy, we observed that inactivity led to a diminished expression levels of PGC-1α, a master regulator of mitochondrial biogenesis and structural/functional integrity both in physiological conditions and during pathophysiological processes of muscle atrophy and aging (Finck and Kelly, 2006). Likewise, we observed a decrease of protein levels for Sirt3, a NAD^+^-dependent protein deacetylase localized solely inside mitochondria (Scarpulla, 2002; Brenmoehl and Hoeflich, 2013), that is known to be a main mitochondrial activity regulator with a prominent role in skeletal muscle (Jing et al., 2011, 2013; Vassilopoulos et al., 2014). Following rehabilitation, the levels of PGC-1α and Sirt3 protein expression rose, consistently with the increased energy needs, and PGC-1α levels reached values even beyond the baseline. Our results are in line with previous data on PGC-1α expression (protein and mRNA) obtained in young subjects (Brocca et al., 2012; Wall et al., 2014). As for Sirt3 data, this is the first study that examined the protein expression levels in relation to bed-rest and subsequent rehabilitation in both Y and E people. Nevertheless, exercise training was documented to upregulate the expression levels of Sirt3 (and PGC-1α) in skeletal muscle by several studies (Lanza et al., 2008; Hokari et al., 2010; Irving et al., 2015). The similar trend exhibited by PGC-1α and Sirt3 in the present study was expected based on the following considerations. (i) PGC-1α in the nucleus, when active, is recognized to regulate Sirt3 expression (Brenmoehl and Hoeflich, 2013); (ii) contractile activity during exercise is documented to trigger signaling pathways leading to Sirt3 induction by PGC-1α (Ventura-Clapier et al., 2008 and references therein); (iii) the overexpression/knockdown of Sirt3 or PGC-1α is reported to elicit in muscle similar effects and to promote the activity of several enzymes involved in oxidative and energetic metabolism (Kong et al., 2010). In addition, Sirt3 can enhance in a positive feedback system PGC-1α expression and the subsequent regulation of mitochondrial related proteins (Palacios et al., 2009; Kong et al., 2010; Hokari et al., 2010; Brenmoehl and Hoeflich, 2013). In line, PGC-1α-Sirt3 signaling pathway triggered by contractile activity is documented to result in both mitochondrial biogenesis and activation of several enzymes of oxidative and energetic metabolism (Palacios et al., 2009; Hokari et al., 2010).

The changes in the expression of such proteins observed in the present study were paralleled by systemic changes (Pišot et al., 2016), with the decrease in response to bed-rest being more pronounced (or statistically significant only) in E and exercise-restoration complete in Y but incomplete in E. Thus, we infer that mitochondrial adaptations occurring under conditions of immobility-induced atrophy, and after exercise training, were associated to PGC-1α-Sirt3 signaling pathway and linked with changes of systemic variables related to mitochondrial function.

### OXPHOS Complexes

Taken as a whole, our data document that the various OXPHOS complexes show diverse patterns of expression following inactivity and rehabilitation, each of them very similar in both E and Y. The patterns of respiratory chain complexes CII, CIII, and CIV, similar to that described for PGC-1α, are in line with studies using a protocol of 2 weeks of one-leg immobilization (Gram et al., 2014). The dissimilar behavior from that of PGC-1α, observed in the cases of CI and CV, may be considered in contrast with the well- known regulation by PGC-1α of the expression of mitochondrial- and nuclear-encoded subunits of OXPHOS (Scarpulla, 2002). Nevertheless, as the observed steady state-levels of proteins are in principle the end-result of biogenesis and degradation, this divergence might be attributed to different responses to immobility and rehabilitation by the degradative pathways of OXPHOS complexes, compared to the expression regulatory pathways linked to PGC-1α. Indeed, it is recognized that disuse muscle atrophy is accompanied by activation of multiple catabolic pathways beside inhibition of protein synthesis (Powers et al., 2012 and references therein – Brocca et al., 2012; Bonaldo and Sandri, 2013; Cannavino et al., 2015). Moreover, PGC-1α might elicit different regulation of single proteins involved in energy production, thereby controlling mitochondrial remodeling rather than biogenesis. Such an effect was described in several reports providing evidence that PGC-1α in skeletal muscle may selectively and differently control the expression levels of several mitochondrial proteins (Chan and Arany, 2014 and references therein).

As for the peculiar behavior exhibited by CV in the experimental conditions of the present study (expression decreased at BR14, but not recovered at R+14), it should be considered that CV is recognized to be finely regulated at post-transcriptional level and to be expressed in large excess with respect to the working molecules. In this context, CV is reported to be a main target of Sirt3, undergoing a deacetylation-mediated activation in several models (Ahn et al., 2008; Bao et al., 2010; Jing et al., 2011; Wu et al., 2013; Lin et al., 2014), and specifically in skeletal muscle in response to exercise-induced stress (Rahman et al., 2014; Vassilopoulos et al., 2014). In this scenario, as Sirt3 expression levels in the present study were documented to be more abundant after rehabilitation both in E and Y, it might be hypothesized that exercise triggered a Sirt3-mediated deacetylation of CV, enhancing the enzyme activity in the presence of unchanged protein expression. These aspects need further investigations.

### Variables Estimating Mitochondrial Mass

The patterns of the expression levels of the mitochondrial matrix protein CS, and of the outer mitochondrial membrane protein TOM20, both usually recognized as reliable mitochondrial mass markers, reveal a marked difference between E and Y subjects, as in these latter no changes were observed. Based on this behavior apparently conflicting with the pattern of PGC-1α, we may hypothesize specific effects on protein turnover as occurring in Y, rather than modulation of mitochondrial biogenesis. Regardless of the mechanism involved in the effects observed, the data of CS and TOM20 expression levels, taken as a whole, are in line with a less pronounced susceptibility to immobility of Y, with respect to E.

### Glycolytic Marker GAPDH

The glycolytic marker GAPDH increased during bed-rest and decreased after rehabilitation in both groups, with a more pronounced effect in Y, suggesting an up-regulation of glycolytic metabolism during bed-rest, possibly as a compensatory response to the mitochondrial impairment, and a subsequent return to a more oxidative metabolism following rehabilitation. These results are part of the still open debate on glycolytic and oxidative metabolism in muscle atrophy and inactivity. In fact, there is not agreement on this topic in literature, likely due to dissimilar protocols applied by diverse authors. In accordance with our data are various bed-rest studies documenting an increased reliance on glycolysis (Acheson et al., 1995; Fitts et al., 2000; Stein and Wade, 2005). Conversely, other reports (Alibegovic et al., 2010b; Moriggi et al., 2010; Ringholm et al., 2011; Brocca et al., 2012) showed a downregulation of both glycolytic and oxidative metabolism during disuse.

### LKB1-AMPK Signaling Pathway

During cell stress events, one of the upstream activators and inducers of PGC-1α expression through phosphorylation is AMPK (Ringholm et al., 2011; Brocca et al., 2012). AMPK is a serine/threonine protein kinase that has emerged as a master sensor of cellular energy balance in mammalian cells, including skeletal myocytes (Hardie et al., 2012), and is recognized to be upregulated by several endogenous stimuli leading to energy impairment, including exercise/muscle contractile activity (Kjøbsted et al., 2018 and references therein). The regulation of AMPK activity is quite complex and, in addition to an allosteric regulation by the [AMP]/[ATP] ratio, it involves also increased phosphorylation by upstream kinases and decreased de-phosphorylation by protein phosphatases. LKB1 appears to be the primary AMPK upstream activating kinase in skeletal muscle under conditions of high-energy stress (Kjøbsted et al., 2018 and references therein). Thus, considering that Ca^2+^/calmodulin-dependent protein kinase kinases (CaMKKs), also key activators of AMPK, were documented to be not considerably expressed in skeletal muscle (Hardie and Sakamoto, 2006), and that CaMKKb was reported to be involved to a lesser extent than LKB1 (Kjøbsted et al., 2018), in the present study we examined the activation of the LKB1-AMPK axis. We determined p-AMPK^Thr172^/AMPK and p-LKB1^Ser482^/LKB1 ratios, and we did not observe any significant change in both groups.

These results suggest that during bed-rest no energy stress (increased [AMP]/[ATP]) was likely present. This is not difficult to conceive. Indeed, skeletal muscle energy turnover in resting conditions is very low, and the energy charge only rarely challenged (Kjøbsted et al., 2018). In addition, the marked decrease in skeletal muscle energy demand during the profound inactivity associated with bed-rest could have played a role even in the presence of downregulated mitochondrial biogenesis and activity. Our results are in accordance with data from another bed-rest study in young subjects (Brocca et al., 2012).

On the other hand, the lack of activation of LKB1-AMPK axis by the rehabilitation intervention could be considered, at a first sight, rather unexpected. According to Combes et al. (2015), high-intensity intervals training (representing the aerobic component of the exercise training regimen adopted in the present study) should elicit pronounced AMPK signaling pathway. This activation is only transient, however, likely as consequence of downregulation or de-phosphorylation of LKB1/AMPK after exercise (Combes et al., 2015). Thus, AMPK activity decreases after exercise to levels observed in resting muscle typically within 3–7 h (Kjøbsted et al., 2018 and references therein). By our protocol, therefore, we might have “missed” the activation of this signaling pathway, due to the interval between the last exercise bout and the muscle biopsy.

In any case, we cannot exclude that the changes of PGC-1α expression, and the resulting modulation of mitochondrial biogenesis/remodeling, observed in the present study were driven by mechanism(s) not linked to LKB1-AMPK axis, among the multiple signaling pathways appearing to converge on regulation of PGC-1α (Gan et al., 2018). One might hypothesize that the decrease in PGC-1α levels observed following inactivity involved Ca^2+^-dependent signaling and was due to diminished intracellular Ca^2+^ levels, which might be counteracted by exercise (Irrcher et al., 2003; Kusuhara et al., 2007; Kang et al., 2012). If this is the case, the greater sensitivity to inactivity observed in E might be explained by the tendency of Ca^2+^ concentration to decrease during aging in skeletal muscle cells (Berchtold et al., 2000 and references therein). A validation of this hypothesis would require additional studies, as alterations of intracellular Ca^2+^ concentration during immobility are still matter of debate due to contrasting reports (Ingalls et al., 1999; Fraysse et al., 2003).

### Gene Expression Analysis

Several studies have tried to comprehend through gene expression analyses the molecular mechanisms involved in skeletal muscle responses to immobility and rehabilitation in humans, as well as associated with aging.

Overall, data emerged from our analysis of GSE24215 gene expression dataset, focused on mitochondria- and OXPHOS-related genes (OXPHOS complexes, PGC1-α, CS) in young adult populations, are in accordance with the results of protein abundance obtained for the young group of our bed-rest study. This support two main messages: (i) decline of the steady-state levels of mitochondria-related proteins in atrophic muscle and recovery after rehabilitation for most of them, (ii) mitochondrial remodeling rather than biogenesis at the basis of mitochondria modulation. Nevertheless, with regard to some discrepancies observed, it should be noted that difference between mRNA and protein stability might be diverse under different conditions. Moreover, the data of gene expression are not always related to the same protein subunits which were analyzed by immunoblot for the single OXPHOS complexes. In the case of complex I, intriguingly, despite NDUFB3 and NDUFB10 gene expression was downregulated by immobility, the absence of changes for NDUFB8, a nuclear DNA-encoded subunit integral to the assembly of complex I, is in accordance with our immunoblot data.

Interestingly, from DEG analysis of GSE24215 dataset we also observed an up- and down-regulation by immobility and exercise training of gene expression for FBXO32 (Atrogin-1) and FBXO2, two essential components of ubiquitin-proteasome pathway. FBXO32 (Atrogin-1) is a specific constituent of muscle playing a critical role in mediating the loss of muscle protein (Lecker et al., 2006). Intriguingly, a more marked upregulation was seen after immobility for FBXO2, which binds to high mannose glycan-containing glycoproteins, and is a gene known to be expressed specifically in the brain. Indeed, as a member of F-box associated family, it displays divergent binding to glycan and glycoproteins, and tissue-specific distributions reflecting differences in glycoprotein distribution (Glenn et al., 2008). In this scenario, upregulation induced by immobility in skeletal muscle is an apparent divergence with respect to tissue specificity of FBXO2. Thus, it is tempting to hypothesize that such rise might reflect a variation of the need for regulation of the myocyte glycome. As mentioned above, increased expression of genes/proteins ascribed to ubiquitin-proteasome pathway is common in atrophic muscle (Reich et al., 2010; Powers et al., 2012 and references therein), and there are data supporting the idea that degradative pathways are enhanced depending on length of immobility (Brocca et al., 2012). Based on these considerations, we may infer that the effects observed in our bed-rest study on the steady-state levels of mitochondria-related proteins might be ascribed, at least in part, to regulation of the expression of ubiquitin-proteasome pathway components.

On the other hand, one must consider also that data from diverse studies should be compared with caution, due to the multiple protocols of immobility and rehabilitative exercise training operated in different laboratories.

From our analysis of GSE8872 dataset emerged that a gene expression downregulation early occurred (5 days of leg cast immobilization) for a number of genes (such as some muscle proteins as MYH3 and MYL12A), including, however, only few mitochondrial OXPHOS subunits. For some of such genes (i.e., NDUFB3, ATP5G3, and MYL12A), a downregulation of the expression resulted also from GSE24215 analysis along with a number of connected genes. GSE8872 data are from *medial gastrocnemius* muscle and comparison between the two datasets was based on similarity to *vastus lateralis* as concerns fiber composition (about 50% fast twitch and 50% slow twitch fibers). The combined data suggest that the immobility effects on mitochondria-related genes appeared to augment with the timespan of the immobility and/or severity on the protocol (leg cast immobilization vs. bed-rest). This finding may be considered in line with our previous reports documenting an impaired mitochondrial respiration, evaluated *ex vivo* in *vastus lateralis* muscle, after prolonged bed-rest conditions (21 days) (Salvadego et al., 2018).

Of note, very few genes from GSE8872 resulted to be significantly upregulated by immobility, among which there is UCP3. An upregulation of muscle UCP3 protein was demonstrated as well, but following prolonged muscle unloading (Mazzatti et al., 2008). In accordance, our recent report proved that after 21 days of bed-rest an enhanced leak respiration (i.e., dissipation of the proton gradient across the inner mitochondrial membrane) occurred associated with a reduced efficiency of OXPHOS (Salvadego et al., 2018). On this basis, we may infer that upregulation of UCP3 gene expression should be an early event in the atrophy program provoked by immobility, in face of the evidence for higher levels of UCP3 protein at later time points upon prolonged immobility conditions (Mazzatti et al., 2008; Salvadego et al., 2018). The relevance of such hypothesis is, in our opinion, in the possibility that uncoupling provoked by immobility would protect the cells against an excessive mitochondrial ROS generation, although at the price of an increased energy dissipation. Indeed, a marked reduction in skeletal muscle energy demand is expected during the profound inactivity of the bed-rest regimen. Comparison with data from GSE24215 dataset showed a significant downregulation of UCP3 gene expression by exercise training, although in this case no effect by immobility was seen.

From another point of view, the finding that MT1X, MT2A appeared also among the few genes, which resulted as significantly upregulated from our analysis of GSE8872 dataset is worthy of note, though not directly linked to mitochondria-related proteins investigated in our bed-rest study. Indeed, metallothioneins are a group of genes associated with muscle atrophy in humans (Lecker et al., 2004) and their increased expression in muscle undergoing atrophy may be necessary to detoxify metals released by metal-containing compounds, such as myoglobin and mitochondrial cytochromes, during muscle protein degradation. In partial accordance with the data emerged from our analysis is an earlier article (Urso et al., 2006) documenting a gene expression upregulation for numerous metallothioneins in *vastus lateralis* muscle after 48 h of knee immobilization. The authors suggested that this may play a role in the initiation of the atrophy program, and inferred that the atrophy program in humans might be denoted by an early transcriptional response for metallothioneins, maybe as consequence of elevated levels of metals and ROS generated in immobilization. Searching in GSE24215 dataset, collecting data from prolonged immobility conditions similar to our bed-rest study, we observed no changes in metallothioneins gene expression in line with the hypothesis that upregulation should be transitory.

Unfortunately, there was not enough tissue remaining from our bed-rest study with which to perform additional assays, but future directions should include measurements for the expression levels of UCP3 and metallothioneins proteins following prolonged immobility, to evaluate their possible involvement in muscle disuse atrophy.

In summary, combining data from the analysis of gene expression of two different datasets with those of protein abundance from our bed-rest study support the idea that immobility and exercise can affect mitochondria-related protein expression levels by both gene expression regulation and protein degradative pathways.

With regard to the expression of mitochondria-related genes involved in aging, by analyzing the GSE9103 dataset (subjects’ age 18–30 and 58–76 years) we focused in particular on certain genes/proteins in *vastus lateralis* relevant for our bed-rest study and obtained some evidence for gene expression downregulation. Specifically, to explain the decline observed for TOM40 and PGC-1α, although it was lower for this latter, it should be postulated that a reduced physical activity by the aged people examined had a crucial role. Indeed, in elderly sedentary subjects exercise was reported to restore PGC-1α protein levels to the ones of young people (Koltai et al., 2012). Furthermore, we have taken advantage from the correlation analysis with age (20–28, 45–55, and 64–75 years) made with data from GSE47881 dataset to obtain information about the behavior of the expression of key mitochondria-related genes. It should be emphasized that the data obtained from the analysis of the two datasets are consistent each other with regard to some, but not all, of such key genes/proteins, specifically, the weak negative correlation with age observed for PPARGC1A should be considered in line with the low decline of PPARGC1A gene expression emerged from GSE9103 analysis. This is also in accordance with the results of our bed-rest study, where at baseline PGC-1α protein abundance was similar regardless of the subjects’ age. It should be considered also that the subjects of the elderly group in our study were moderately active, differently from sedentary people whose data of gene expression were used to create both GSE9103 and GSE47881 datasets. Concerning gene expression for OXPHOS complexes’ subunits, negligible changes were observed in GSE9103 dataset, in apparent conflict with the results emerged from the correlation analysis made with GSE47881 data. This may suggest that a more advanced age should be needed to elicit appreciable downregulation of such genes. In accordance with this hypothesis are data obtained at baseline in our bed-rest study where the OXPHOS protein abundance investigated was similar regardless of the subjects’ age, except for complex CII that was higher in the elderly subjects. However, the downregulation of gene expression for TMEM70 and COA7 observed in GSE9103 dataset reminds to the strong negative correlation of NDUFAF6, COA4, and UQCC3 emerged from GSE47881 dataset analysis. Downregulation of such genes during aging should be taken into account when one evaluate the similar protein expression levels for single subunits of complexes I, III, IV, and V observed at baseline in our bed-rest study for E and Y groups. Indeed, such genes encode for assembly factors of ATP synthase complex V and of respiratory chain complexes I, III, and IV; thus their decline might be responsible to reduce in elderly subjects the assembly of whole complexes in membrane. However, future studies will need to address this.

In conclusion, data from our analyses of the effects of aging on expression of a number of mitochondria-related genes relevant in the context of the proteins analyzed in our bed-rest study prompt us to highlight that immobility should be more critical for mitochondrial efficiency and energy production in case of people more aged than our elderly subjects. Indeed, we might infer that the trend of the effects documented by our bed-rest study should go on with age.

### Study Limitations

The limited number of subjects enrolled in our study may weaken its outcomes. Nevertheless, this is a consequence of logistical limitations, which are intrinsic to this type of studies. Specifically, this is a very complex study performed with two populations of subjects (Y and E) and three muscle biopsies per subject, one of them obtained after 14 days of bed-rest and another one after 14 days of supervised exercise program. From a statistical standpoint, limitations are mainly related to the uneven sample size of the groups (7 Y and 16 E subjects) and particularly to the more restricted size of Y group that impact on statistical power to detect differences at some points.

In addition, only limited amounts of tissue specimens from the muscle biopsies were available for our experiments, due to the numerous participants to the bed-rest campaign where our study was comprised. This reduced the number of proteins we decided to assay by quantitative immunoblot analyses in order to achieve accurate quantifications. Most important in this respect, we did not determine the expression levels of key components of ubiquitin-proteasome pathway despite our hypothesis that a reduction of mitochondrial-related proteins in atrophic muscle might be ascribed, at least in part, to regulation of the expression of such pathway. Our hypothesis is validated based on our gene expression data mining from human public datasets, as well as on solid literature.

## Conclusion

In summary, based on the expression levels of key proteins related to mitochondrial biogenesis regulation and bioenergetics in *vastus lateralis* muscle, our study confirms a crucial role of mitochondrial biogenesis/remodeling in muscle plasticity following inactivity and exercise rehabilitation. The heterogeneous patterns of the expression levels observed for some proteins are indicative of different responses to the treatments by the respective degradative ways compared to the biogenesis regulatory pathways. Furthermore, our study provide evidence that responses to bed-rest causing atrophy, as well as adaptations to rehabilitation, in E and Y populations were of different extent and qualitative diverse. Namely, the impact of bed-rest of most proteins was greater, and the rehabilitation recovery was less complete in the elderly subjects, where the changes observed were associated with modifications of mitochondrial mass. Results on protein expression levels are reinforced by data obtained from *in silico* analyses of four public human gene expression datasets, focusing on mitochondria-related genes affected in skeletal muscle responses to disuse and rehabilitation of adult individuals, or declined in association with aging.

## Datasets analyzed in the study

Alibegovic, A, C., Sonne, M.‘P., Højbjerre, L., Bork-Jensen, J., Jacobsen, S., Nilsson, E., Frch, K., Hiscock, N., Mortensen, B., Friedrichsen, M., Stallknecht, B., Dela, F., Vaag, A. Sep 20, 2010. Insulin resistance induced by physical inactivity is associated with multiple transcriptional changes in skeletal muscle in young men. GEO – Gene Expression Omnibus, version May 15, 2017 [GSE24215].

Asmann, Y. W., Nair, K. S., Lanza, I. R., Short, D. K., Short, K. R., Bigelow, M. L., Joyner, M. J. submission date sep 19, 2007. Skeletal Muscle Transcript Profiles in Trained or Sedentary Young and Old Subjects. GEO – Gene Expression Omnibus, version Oct 29, 2018, [GSE9103] last update date Dec 10 2018.

Phillips, B. E., Timmons, J. A. Jun 12, 2013. Impact of resistance exercise on human skeletal muscle gene expression – ageing. GEO – Gene Expression Omnibus, version Oct 29, 2018 [GSE47881].

Yiwen, C. Aug 24, 2007. Transcriptional pathways associated with skeletal muscle disuse atrophy in humans. GEO – Gene Expression Omnibus, version Jul 08, 2016 [GSE8872].

## Ethics Statement

This study was performed in accordance with the ethical standards of the 1964 Declaration of Helsinki and was approved by the National Ethical Committee of the Slovenian Ministry of Health on April 17, 2012 under the acronym: IR-aging 1200.

## Author Contributions

BG and IM designed the research. DS, BŠ, RadP, and JR organized the bed-rest campaign. AB and BM performed the experiments, prepared figures, and references’ list. AB and MC analyzed the data. RafP performed the gene expression analyses. MI performed the statistical analysis. AB, MC, and IM interpreted the results of experiments. IM, MC, AB, and BG edited and revised the manuscript. All authors approved the final version of manuscript.

## Conflict of Interest Statement

The authors declare that the research was conducted in the absence of any commercial or financial relationships that could be construed as a potential conflict of interest.

## Footnotes

1 http://brainarray.mbni.med.umich.edu/Brainarray/Database/CustomCDF/genomic_curated_CDF.asp

## Abbreviations

AMPK: AMP-activated protein kinase
ATP5A1: ATP synthase F1 subunit alpha, mitochondrial complex V
BDC: baseline data collection
BSA: bovine serum albumin
CaMK: calcium/calmodulin-dependent protein kinase
CaMKKs: calcium/calmodulin-dependent protein kinase kinases
COA4: cytochrome c oxidase assembly factor 4 homolog
COA7: cytochrome c oxidase assembly factor 7
COX IV: cytochrome c oxidase subunit 4, mitochondrial complex IV
COX7C: cytochrome c oxidase subunit 7C
CS: Citrate Synthase
DEG: differentially expressed genes
FBXO32: atrogin-1
GAPDH: glyceraldehyde-3-phosphate dehydrogenase
GPD2: glycerol-3-phosphate dehydrogenase 2
LKB1: Liver kinase B1
NDUFA8: NADH:ubiquinone oxidoreductase subunit A8
NDUFAF6: NADH:ubiquinone oxidoreductase complex assembly factor 6
NDUFB8: NADH dehydrogenase [ubiquinone] 1 beta subcomplex subunit 8, mitochondrial complex I
NDUFC1: NADH:ubiquinone oxidoreductase subunit C1
OXPHOS: oxidative phosphorylation
PGC-1α: Peroxisome proliferator activated receptor-γ coactivator-1α
PVD: polyvinylidene fluoride
ROS: reactive oxygen species
SDHB: succinate dehydrogenase [ubiquinone] iron-sulfur subunit B, mitochondrial complex II
Sirt3: Silent mating-type information regulation 2 homolog sirtuin 3
TIMM23: translocase of inner mitochondrial membrane 23
TIMMDC: translocase of inner mitochondrial membrane domain containing 1
TMEM70: transmembrane protein 70
TOM20: mitochondrial import receptor subunit TOMM20 homolog
TOMM40L: translocase of outer mitochondrial membrane 40 like
Tris: tris(hydroxymethyl)aminomethane
UCP3: uncoupling protein 3 - mitochondrial proton carrier
UQCC3: ubiquinol-cytochrome c reductase complex assembly factor 3
UQCR11: ubiquinol-cytochrome c reductase complex III subunit XI
UQCRC2: cytochrome b-c1 complex subunit 2, mitochondrial complex III
