# Supplementary material for: Mitochondrial Adaptations in Elderly and Young Men Skeletal Muscle Following 2 Weeks of Bed Rest and Rehabilitation
Source: Front Physiol. 2019 May 1;10:474. doi: 10.3389/fphys.2019.00474 (PMC6504794; doi:10.3389/fphys.2019.00474)
Supplement: Supplementary file 6 [file Data_Sheet_1.docx]

**SUPPLEMENTARY TABLE LEGENDS**

**Supplementary Table 1. List of the top differentially expressed genes in skeletal muscle following inactivity, from GEO dataset GSE24215**.

Data analyzed are from *vastus lateralis* muscle biopsies of healthy adult (24-27 years old) volunteers subjected to complete inactivity due to bed-rest. DEG analysis was carried out in relation to data obtained from the same subjects before inactivity taken as control values (ctrl). Figures represent ctrl/inactivity ratios (cut-off FC 1.5, p < 0.05).

**Supplementary Table 2. List of the top differentially expressed genes in skeletal muscle following post-inactivity exercise rehabilitation, from GEO dataset GSE24215**.

Data analyzed are from *vastus lateralis* muscle biopsies of healthy adult (24-27 years old) volunteers subjected to exercise rehabilitation after complete inactivity due to bed-rest. DEG analysis was carried out in relation to data obtained from the same subjects before exercise. Figures represent inactivity/exercise ratios (cut-off FC 1.5, p < 0.05).

**Supplementary Table 3. List of the top differentially expressed genes in skeletal muscle following cast immobilization, from GEO dataset GSE8872.**

Data analyzed are from *medial gastrocnemius* muscle biopsies of healthy adult (around 30 years old) volunteers subjected to short (5 days) leg cast immobilization. DEG analysis was carried out in relation to data obtained from the same subjects before immobilization taken as control values (ctrl). Figures represent ctrl/immobility ratios (cut-off FC 1.5, p < 0.05).

**Supplementary Table 4. List of the skeletal muscle top differentially expressed genes in aged** **people, from GEO dataset GSE9103.**

Data analyzed are from *vastus lateralis* muscle biopsies of healthy young (18-30 years) and old (58-76 years) sedentary subjects. Figures from DEG analysis represent old/young ratios (cut-off FC 1.3, p < 0.05).

**Supplementary Table 5. Negative correlated genes with age in skeletal muscle, from GEO dataset** in **GSE47881**

Data analyzed are from *vastus lateralis* muscle biopsies of tree different groups of sedentary subjects (age 20-28, 45-55 and 64-75 years). Figures represent rule of thumb values for genes with a strong negative correlation with age (cut-off correlation coefficient -0.6, p < 0.05).
